# Rigid crosslinker-assisted nondestructive direct photolithograph for patterned QLED displays

**DOI:** 10.1038/s41377-025-01918-7

**Published:** 2025-07-24

**Authors:** Zhong Chen, Zhongwei Man, Shichao Rao, Jinxing Zhao, Shuaibing Wang, Runtong Zhang, Feng Teng, Aiwei Tang

**Affiliations:** Key Laboratory of Luminescence and Optical Information, Ministry of Education, School of Physical Science and Engineering, Beijing Jiaotong University, Beijing, 100044 China

**Keywords:** Inorganic LEDs, Quantum dots

## Abstract

Recently, colloidal quantum dots (QDs) with high luminescent efficiency and tunable colors have become ideal materials for next-generation display devices. Direct photolithography is a powerful tool for patterning QD devices, but it faces the serious issue of degradation in the photophysical properties of the patterned QDs. Here, we use relatively rigid cyclopentane as a bridging group to design the crosslinker CPTA, achieving high-resolution direct photolithography of QDs with nearly nondestructive under ambient conditions. The key to the crosslinker design is the introduction of a rigid bridging group that elevates the LUMO level, providing a stronger energy barrier to prevent QD electrons from being trapped or undergoing non-radiative recombination, thus preserving their PL and EL properties. The efficient and high-resolution RGB line and dot arrays were fabricated with pixel sizes down to 1 μm and a resolution of up to 6350 PPI. The patterned RGB QD films, especially red QDs, maintained their optical and optoelectronic properties, with patterned red QLEDs achieving peak external quantum efficiency (EQE) of 21% and a maximum luminance (*L*_max_) of ~180,000 cd m⁻², matching pristine devices. These results highlight the importance of photo-crosslinker design for nondestructive QDs patterning, paving the way for advanced display applications in patterned QLED technology.

## Abstract (web summary)

This work innovatively designed a rigid high-LUMO bridging photo-crosslinker for nondestructive high-resolution QD patterns with a resolution of 6350 PPI, and high-performance QLED displays with EQE of 21%.

## Graphical abstract

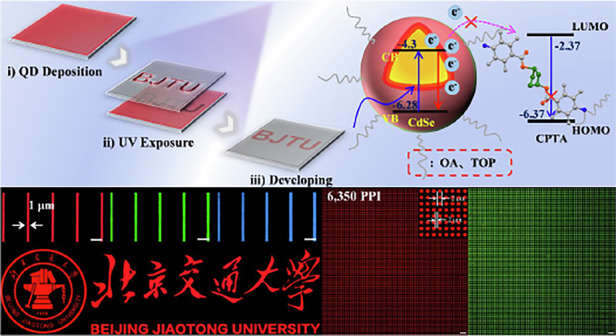

## Introduction

Colloidal quantum dots (QDs) are chemically synthesized materials with size-dependent tunable optical features, such as narrow-band emission, high luminescent efficiency and a wide color gamut, making them highly suitable for next-generation display devices^1–6^. However, with the advancement of the device fabrication processes, the growing demands for pixelated and information density in highly integrated photonic devices necessitate QDs capable of being patterned at microscales^7–10^. To achieve this goal, patterning technologies represented by inkjet printing^11–13^, transfer printing^14–18^, and traditional photolithography^7,8,19–24^ have been validated and developed. However, the challenge with these technologies is the difficulty achieving a balance between patterning yield, resolution, chemical compatibility with QDs as well as device dependency^25–32^. Typical for conventional photolithography, the structure of QD materials is frequently damaged by energy loss and chemical environment effects during the photoreaction process, leading to reduced patterning accuracy and compromised optical performance^7,8,21,23,24,29^. Consequently, developing novel patterning techniques that can achieve higher resolution while maintaining the stability of the optical and electrical properties of the material has become a critical focus in the advancement of QD display technology.

Alternatively, direct lithography technology eliminates the need for photoresists, streamlines the process, avoids the complex interactions between photoresists and QDs, and mitigates the negative effects of etching on performance, making it a promising candidate for patterning methods^28,29,33–42^. In principle, direct lithography leverages the photosensitivity of QDs in combination with photosensitive crosslinkers, during exposure, the QD and photosensitive compound mixture undergoes a series of photochemical reactions, enabling the selective removal of the more soluble regions with a developer, thereby creating the desired QD patterns^43,44^. Leveraging this mechanism, crosslinkers with azide or azo groups as photoreactive sits have shown outstanding performance in patterning technology^44–49^. More importantly, by omitting the photoresist layer, QDs can be uniformly deposited on the substrate, facilitating the formation of high-resolution patterns.

It is worth noting that even the state-of-the-art crosslinkers available today cannot fully prevent photodamage to the photoluminescence (PL) and electroluminescence (EL) of QDs due to photochemical reactions and surface ligand modifications^29,50,51^. Due to the lack of an effective π-conjugated structure, the main absorption peak of the crosslinks is typically below 300 nm, primarily originating from benzene ring absorption. As a result, 254 nm deep ultraviolet (DUV) light has become the wavelength of choice for most current photolithography crosslinker, although some crosslinkers have been developed for use with 365 nm exposure. However, intense DUV exposure inevitably causes damage to the QD surface, leading to the formation of trap states, which significantly reduces the photoluminescence quantum yield (PLQY) of the QDs. According to reports, these factors can typically lead to a 30-40% reduction in the PLQY of QDs patterned with azide-based crosslinkers (Fig. S1)^44,45,49^, significantly impacting pattern quality and device efficiency. In addition, electronic structures of the crosslinker are another key factor contributing to the reduction in PLQY. For example, when the lowest unoccupied molecular orbital (LUMO) energy level of the crosslinker is lower than the conduction band of the QDs, the excited-state electrons of the QDs will inevitably transfer to the crosslinker through the surface ligands of the QDs, resulting in energy loss. In contrast, elevating the LUMO energy level of the crosslinker helps raise the conduction band of the crosslinked QDs, thereby reducing the electron transfer rate, balancing electron-hole recombination, and ultimately improving device efficiency and stability. Therefore, enhancing the light absorption capacity of crosslinkers to reduce the demand for DUV exposure, as well as elevating the LUMO energy level of crosslinkers to minimize charge transfer, are effective ways to address the reduction in PLQY. While some researchers have begun exploring this area, the challenge persists, and effective strategies are still needed to address it^47^.

Here, we propose a rigid crosslinker design strategy that reduces the light dosage requirement and raises the LUMO energy level, enabling nearly nondestructive direct photolithography under ambient conditions. Rigid-designed crosslinker (cyclopentane-1,3-diyl bis(4-azido-2,3,5,6-tetrafluorobenzoate), CPTA) enables high yield QDs patterning under low DUV dosage (20 mJ cm^−2^). As the results, 1 μm high-resolution line (up to 6,350 PPI dot matrix) and full-color patterns of QDs can be fabricated without affecting their optical and electrical properties. QLEDs with patterned red, green, and blue QD layers were fabricated, with the red device achieving a peak EQE of ∼21% and a maximum luminance (*L*_max_) of ∼ 180,000 cd m^−2^, matching the performance of the pristine devices.

## Results

### Design of the rigid crosslinker

**Fig. 1:**
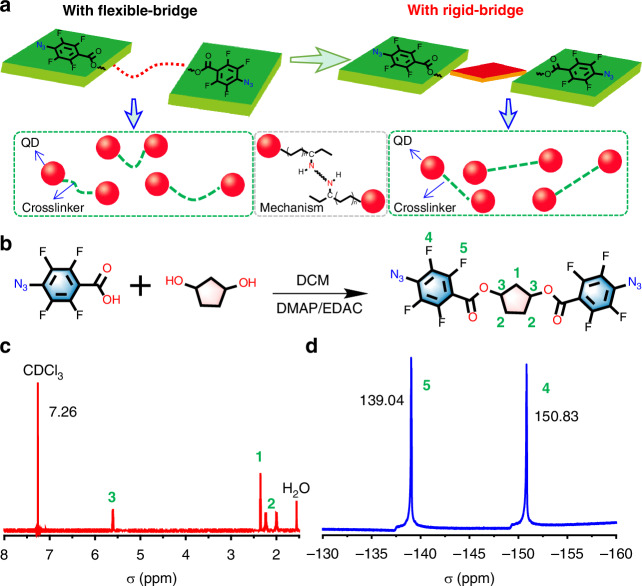
Design and synthesize rigid crosslinker of CPTA. **a** Comparison of flexible and rigid crosslinkers and the mechanism of azide-based crosslinkers in QDs crosslinking. **b** Synthesis route and molecular structure of CPTA. **c** ^1^H NMR and **d** ^19^F NMR spectra of CPTA

The currently reported crosslinkers, typically featuring bridging segments made of flexible groups with varying chain lengths such as ethylene glycol, lead to uneven distances between QDs after coupling and only a modest reduction in solubility, necessitating high UV dosage and prolonged UV exposure (Fig. 1a). The challenge lies in the significant decline in the PL and EL stability of the QDs. Therefore, the development of molecular systems, particularly the bridging groups, is imperative. Cyclopentane (CPT) is one of the simplest cycloalkanes, consisting of a five-membered carbon ring, with high-octane and knock-resistant properties, and is commonly found in commercial gasoline. Compared to alkanes, CPT exhibits a certain degree of rigidity^52^. Crosslinker with CPT as the bridging group shows restricted rotation, with a length of 2 nm (Fig. S2). Importantly, the rigidity of CPT leads to a remarkable decrease in QDs solubility after cross-linking, which helps reduce the DUV exposure requirements. Following above design, we chose CPT as the rigid bridging group and the traditional azide groups the photoactive site for QD patterning. CPTA was prepared by adapting a published procedure involving the treatment of 4-azido-2,3,5,6-tetrafluorobenzoic acid with dichloromethane and 1,3-cyclopentanediol in the presence of DMAP and EDAC at room temperatures (Fig. 1b). The product was purified by column chromatography, followed by recrystallization in dichloromethane (DCM)/*n*-hexane, and its purity was confirm using HPLC (Fig. S4). In addition, the chemical structure featured by ^1^H NMR, ^19^F NMR, high-resolution mass spectroscopy (HRMS) and single crystal X-ray diffraction (CCDC number is 2418785) (Fig. 1c, d, Fig. S3 and Table S1).

### Rigid crosslinker for patterning

**Fig. 2:**
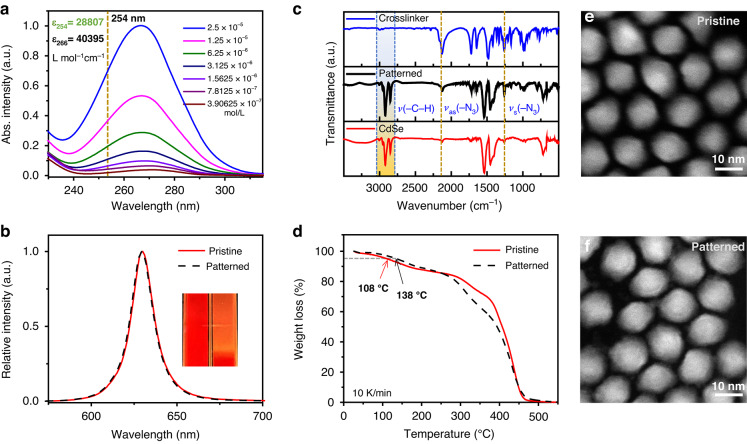
Optical and morphological characterization of red QDs before and after crosslinking. **a** Absorption spectra of CPTA in DCM solvent with different concentrations. **b** Emission spectra of red QDs before and after crosslinking. Inset is the images of QDs before and after crosslinking in *n*-octane solution. **c** FTIR spectra of CPTA, pristine QD films and patterned films. **d** TGA analysis of red QDs before and after crosslinking. HRTEM of **e** pristine and **f** patterned red QDs

The strong UV absorption ability can reduce light dosage requirement, benefiting for efficient and non-destructive patterning. As shown in Fig. 2a, the maximum absorption peak of CPTA is located at 266 nm, which is very close to the commonly used 254 nm DUV light. The molar extinction coefficients (ε) at 266 nm and 254 nm are 40,395 and 28,807 L mol^−1^ cm^−1^, respectively, and the high ε ensures that the crosslinker undergoes sufficient photochemical reactions without disrupting the process. Figure 2b shows the emission spectra of QDs before and after the addition of the crosslinker. As can be seen, the emission peak and CIE coordinates of the QDs remains unchanged (Fig. S5), indicating that the crosslinker did not significantly affect the emission properties of QDs. In addition, the QD n-octane solution with the addition of CPTA exhibits significant precipitation after UV exposure, indicating that a photochemical reaction occurred between the crosslinker and the QDs, leading to a decrease in solubility and potential for achieving phototherapy. Figure 2c presents the Fourier transformed infrared (FTIR) spectra of the crosslinker, QDs, and the crosslinked composite material. Prior to crosslinking, the crosslinker and QDs display their respective characteristic absorption peaks. After crosslinking, some of these peaks shift or disappear, particularly those corresponding to C-H bonds (∼2800–3050 cm^−1^) and azide groups (N₃, ∼2100 cm^−1^). This indicates that the crosslinker underwent photochemical reactions with the ligands on the QD surface, resulting in the formation of crosslinked structures. Thermogravimetric testing (Fig. 2d) shows that the T_95_ of the patterned samples increased from 108 °C to 138 °C, further confirming the occurrence of crosslinking, and also indicating an improvement in thermal stability, which is beneficial for device applications. The photophysical properties of QD depend on their size and morphology. High-resolution TEM images indicate that there are no significant size or morphology changes before and after crosslinking (Fig. 2e, f). Notably, the QDs surface ligands employed are trioctylphosphine (TOP) and oleic acid (OA), both of which lack nitrogen (N) atoms (Fig. S6). After crosslinking, the QD surfaces are enriched with N element, and fluorine (F) elements are widely distributed in the pores between the QDs (Fig. S7), further indicating that a photoactivated reaction occurred between the crosslinking agent and the QDs.

**Fig. 3:**
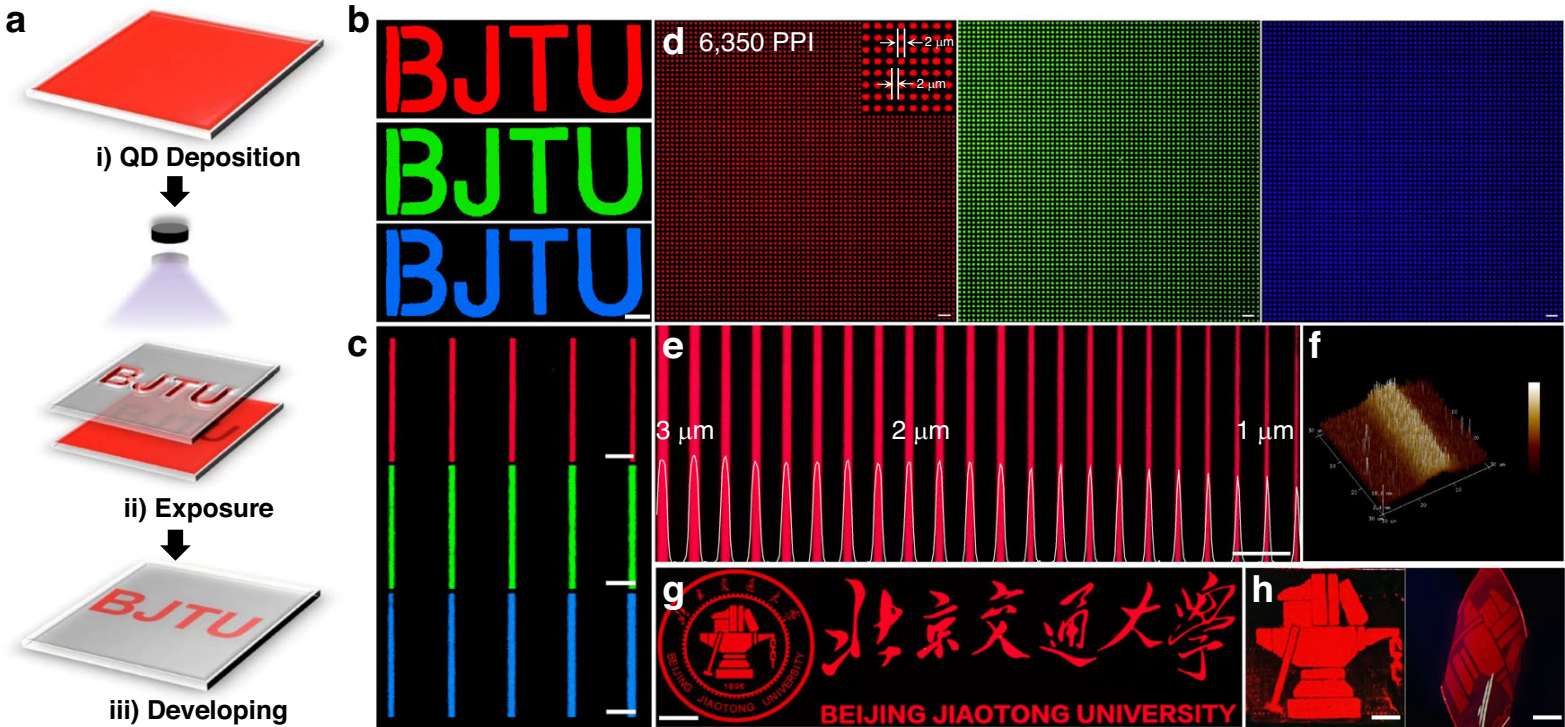
Optical and microscopic images of patterned QD. **a** Schematic diagram for patterning procedures. Fluorescent microscopic images of red, green, and blue **b** “BJTU”, **c** uniform 1 μm line arrays, **d** 2 μm dot arrays and **e** line patterns (width, 1-3 μm). **f** AFM image and the height profile of QD line patterns with a width of 10 μm. Fluorescent microscopic images of patterned QDs in the shape of Beijing Jiaotong University logo under **g** rigid and **h** flexible PET substrates. Scale bars: **b** 2 mm, **c** 5 μm, **d** 10 μm, **e** 20 μm, **g** 500 μm, and **h** 2 mm

Figure 3a illustrates the standard QD lithography process, which comprise three key steps: (i) QD Deposition, where a uniform layer of QD material is deposited onto the substrate. (ii) Exposure, during the UV light expose the QDs, initiating a photochemical reaction between the QDs and the crosslinker, resulting in the formation of crosslinked structures. (iii) Developing, where a developing solvent removes the non-crosslinked QD regions, leaving only the crosslinked areas and creating a precise pattern on the substrate. The specific operational details are described in the supporting information. Through the above steps, we successfully achieved the tricolor patterning of the abbreviation for Beijing Jiaotong University (BJTU) using blue, green and red QDs with a “BJTU” mask (Fig. 3b). It is worth noting that, thanks to the significant difference in solubility before and after crosslinking brought by the rigid CPTA, the patterned background is exceptionally clean with a high patterning yield (90%). Subsequently, we used a mask with the equal width and spacing of 10 μm each to evaluate the quality of the patterning. As shown in Fig. S8, the patterned stripe width matched that of the mask, demonstrating that the lithography process maintained a high degree of consistency even at larger scales.

To investigate the imaging resolution capability of the our crosslinker, we conducted studies on pixelated arrays and line patterns. As shown in Fig. 3c, the finest line width is 1 μm, reaching the resolution limit of the mask, which is comparable to the state-of-the-art direct lithography crosslinking agents but requires only half the light exposure dose reported in the literature^44^. Moreover, the pixelated three-primary-color arrays can achieve pixel sizes of the 2 μm with a spacing distance of 2 μm, corresponding to a resolution of ~6350 PPI (Fig. 3d). Figure 3e displays a gradient line pattern, narrowing from 3 μm to 1 μm. This gradient pattern demonstrates the lithography process’s ability to precisely control line width variations, enabling the creation of structures of varying sizes on the same substrate. This flexibility is crucial for adjusting electrical and optical properties, particularly in optoelectronic devices with functional gradients. The height distribution and morphology of each line width region were measured using grayscale images processed with Image J (Fig. 3e). The height measurements clearly show the precise width and accurate distribution of each line, highlighting the lithography process’s consistent control across different line widths, ensuring both precision and stability (Fig. S9). Atomic force microscopy (AFM) (Fig. S10) images provide a direct comparison of the quality between pristine and patterned QD films, showing no changes in morphology, thickness, and surface roughness (∼1.8 nm). Additionally, the QDs remain tightly arranged with no noticeable pinholes or cracks, indicating that the lithography process can precisely control both the in-plane line width and the thickness and shape of the lines. Furthermore, even with only 10 wt% crosslinker and an exposure dose of 20 mJ cm⁻², the QD film exhibited a high retention rate (80%) along with excellent pattern fidelity (Fig. S12), which contributes to the preservation of their PL and EL performance.

We also realized that CPTA can perform photolithography in air, which is very difficult to achieve, as the activated product arylnitrene is easily quenched by oxygen and water^44^. This advantage will also expand the applicability of CPTA in more environments. Subsequently, we investigated the imaging capability of the CPTA for complex patterns at the micron scale in air. As shown in the Fig. 3g, the “Beijing Jiaotong University” logo and the artistic Chinese characters are displayed very clearly, with no interconnection or blurring, even at the turning points. Besides patterning on rigid substrates, this method can also be well integrated with flexible substrate, which is expected to enable the fabrication of flexible and wearable devices. For example, high-fidelity QD patterns can also be achieved on a poly(ethylene terephthalate) (PET) substrate following the same operational steps (Fig. 3h).

**Fig. 4:**
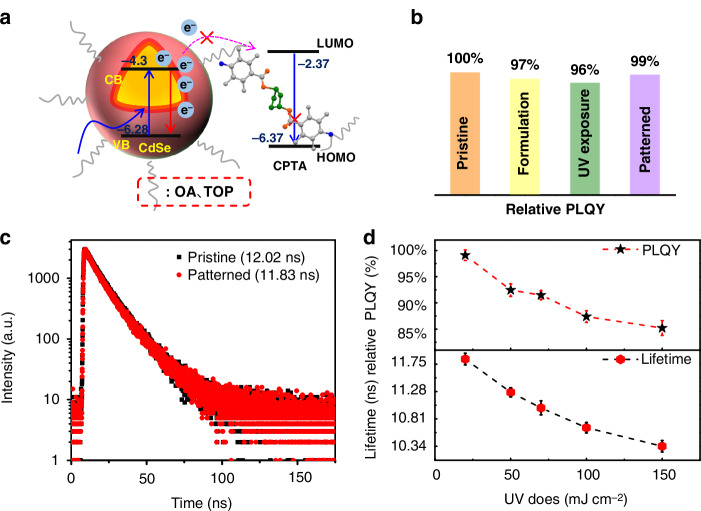
PL characteristics of patterned QDs. **a** Schematic energy Level of red QDs and crosslinker CPTA. **b** Relative PLQYs of QD films at various stages of patterning: Pristine, formulation (QDs + 10 wt% of CPTA), UV exposure (20 mJ cm^−2^), and patterned. **c** Fluorescence decay profiles of pristine and patterned QD films. **d** the relative PLQYs and lifetimes for QD films with CPTA at different doses

The crosslinker possesses appropriate electronic energy levels and excellent photochemical properties, which are beneficial for achieving improved PL and EL characteristics in patterned QDs and related devices. Therefore, we investigated the energy level structures and photostability (PLQY and lifetime) before and after crosslinking. The highest occupied molecular orbital (HOMO) and the lowest unoccupied molecular orbital (LUMO) of CPTA, QD and patterned samples can be estimated using a combination of ultraviolet photoelectron spectroscopy (UPS) and ultraviolet/visible absorption spectra (Fig. S13). Based on the test results, a schematic diagram of the electronic energy alignment was drawn in Fig. 4a. Clearly, the HOMO and LUMO energy levels of CPTA are located outside those of QDs, indicating that CPTA cannot accept photogenerated carriers (electrons) from the QDs. In other words, the higher HOMO energy provides a stronger energy barrier for QD electrons, preventing them from being trapped or following non-radiative recombination pathways after light absorption. This further validates the effectiveness of the strategy of rigid structure design in limiting the range of electron delocalization and thereby elevating the LUMO energy level. Subsequently, to verify the superiority of this energy level structure, we monitored the PLQY (Fig. 4b) and steady-state lifetime (τ) of the QD film at different stages of the patterned process. These stages include the pristine (film containing only QDs, with an absolute PLQY of 42.1%, defined as 100% relative), UV exposure (film containing only QDs after 20 mJ cm^−2^ irradiation), formulation (film containing QDs and crosslinker, unexposed), and patterned (film after photochemical crosslinking and development). QD film remains 97% of its PLQY after expose to 20 mJ cm^−2^ for 5 min at 254 nm. However, the PLQY slightly increased to 97% after the addition of the CPTA and even retained 99% after patterning. This indicates that CPTA effectively suppresses surface defects of QDs and compensates for the inherent damage caused by DUV exposure.

In addition, Fig. 4c compares the fluorescence lifetimes of pristine and patterned QDs films. The pristine QDs film exhibits a fluorescence lifetime of 12.02 ns, while the patterned film shows a slight reduction to 11.83 ns. The slight decrease in lifetime is negligible and falls within the margin of error, indicating that the lithography process does not significantly affect the luminescence characteristics of the QDs. Moreover, we investigated the variation of the PLQY and τ of patterned QDs with the dose of 254 nm DUV (Fig. 4d). With prolonged UV irradiation, the PLQYs and lifetimes of QDs after complex patterning decreased. However, even under 150 mJ cm^−2^ DUV exposure, >85% of the PLQY could be retained, attributed to the stronger energy barrier effect of CPTA. The calculations revealed a notable increase in the non-radiative decay rate, suggesting that the decline in PLQY is primarily attributed to the increased surface defect density of the QDs (Table S2). Notably, a DUV dose of 20 mJ cm^−2^ is sufficient to meet the exposure requirements, achieving nearly nondestructive lithography. Maintaining such a high PLQY is crucial for ensuring both the efficiency and long-term stability of display devices.

**Fig. 5:**
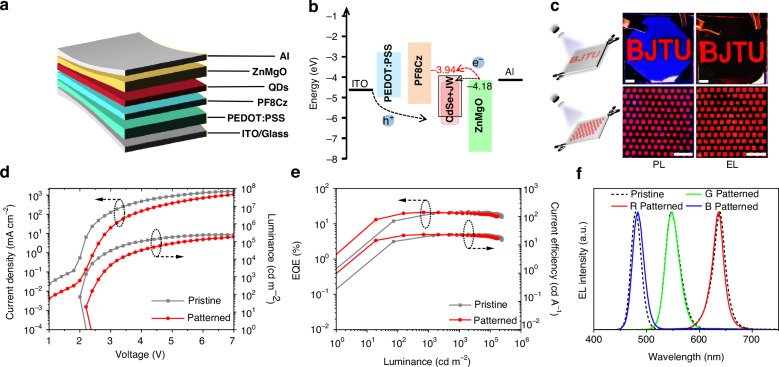
Device performance of pristine and patterned QLEDs. **a** QLED device structure and **b** Corresponding energy level diagram. **c** PL (left) and EL (right) images of the patterned red QLED device. Scale bars 3 mm. **d** *J-V-L* and **e** *EQE-CE-L* characteristics of pristine and patterned red devices. **f** The EL (solid line) and PL (dash line) spectra of the RGB devices

The nondestructive lithographic process encourages us to fabricate electroluminescent patterned QLED devices. The device structure consists of ITO, PEDOT: PSS, PF8Cz, patterned QDs, ZnMgO, and aluminum (Fig. 5a). Figure 5b illustrates the energy level diagram of the functional layers in the QLED device. It is evident that the energy levels of the patterned QDs are elevated, which helps reduce the electron injection rate, thereby compensating for the non-radiative recombination caused by exposure and maintaining the device performance. To achieve array formation, a thin PMMA layer was inserted between the pattern QD layer and ZnMgO, enabling electroluminescent patterning and resulting in clear, well-defined “BJTU” and 500-micron square arrays (Fig. 5c). First, we compared the EL performance of the pristine and patterned (non-arrayed) red QD devices, with the corresponding data recorded in Table 1. By comparing the current density-voltage-luminance (*J*-*V*-*L*) characteristics of the pristine and patterned devices (Fig. 5d), it can be observed that the patterned device has a slightly higher turn-on voltage (V_on_), increasing from 2 V to 2.4 V, due to the elevation of the patterned QDs’ energy level. The maximum luminance decreased from 240,000 cd m^−2^ to 180,000 cd m^−2^. Additionally, both the pristine and patterned devices demonstrated outstanding performance, achieving EQE exceeding 21%, with the patterned device retaining 96% of the pristine device’s EQE and 98% of its current efficiency (CE_max_) (Fig. 5e). Furthermore, the T_95_ of the patterned device at 10,000 nit was slightly higher than that of the pristine device (Fig. S14), indicating excellent operational stability. The EL peak wavelength and width remained unchanged (Fig. 5f). Of course, the CPTA crosslinker can also function in green and blue devices, but the efficiency, particularly for blue devices, decreases significantly (Table 1 and Fig. S15). This may be attributed to injection imbalances caused by changes in the energy level structure. These results highlight the great potential of QD and crosslinker integration for electroluminescent applications, providing crucial support for the development of high-performance and high-definition display devices in the future.

**Table 1:** Device performances of pristine and patterned RGB QLEDs

QDs	Status	V_on_ (V)	*L*_max_ (cd m^−2^)	EQE_max_ (%)	CE_max_ (cd A^−1^)
Red	Pristine	2.0	241,844	21.99	23.63
	Patterned	2.4	179,872	21.08	23.27
Green	Pristine	2.4	436,033	11.99	55.70
	Patterned	2.4	244,884	9.59	40.92
Blue	Pristine	2.6	9079	3.92	3.22
	Patterned	3.0	4939	1.95	1.47

## Discussion

In summary, we designed a crosslinker with a rigid cyclopentane-based bridging group, achieving damage-free photolithography under ambient conditions and validating its application in arrayed high-performance QLEDs. The rigid structure design elevated the LUMO energy level of the crosslinker, effectively suppressing carrier and exciton trapping. As a result, the photophysical properties before and after photolithography remained almost unchanged. The resulting line arrays exhibit pixel sizes down to ∼ 1 μm and resolutions up to 6,350 PPI. In addition, the crosslinker can be applied on PET flexible substrates. QLED pattern tests revealed that CPTA can be applied to blue, green, and red QDs, particularly for red QDs, where the EL performance (with EQE >21% and *L*_max_ exceeding 180,000 cd m^−2^) showed no significant degradation. Our findings suggest that the limitations of direct photolithography in QLED manufacturing can be mitigated through strategic material design. Our work provides a promising pathway for the development of next-generation high-efficiency, high-resolution displays using direct photolithography.

## Materials and methods

### Materials

4-Azido-2,3,5,6-tetrafluorobenzoic Acid (C_7_HF_4_N_3_O_2_, ≥98%, Aladdin), 4-Dimethylaminopyridine (DMAP, Shanghai yuanye Bio-Technology), 1,3-Cyclopentanediol (Bidepharm), N_1_-((Ethylimino)methylene)-N_3_,N_3_-dimethylpropane-1,3-diamine xhydrochloride (EDAC, Bidepharm), Dichloromethane (Innochem), Magnesium sulfate (Aladdin), red, green, and blue CdSe QDs were purchased from Hefei Funa Technology Co., Ltd. Other solvents were purchased from Beijing TongGuang Fine Chemicals Company.

### Synthesis of CPTA

In a typical synthesis, weigh 1.42 mmol (334 mg) of 4-azido-2,3,5,6-tetrafluorobenzoic acid and 0.147 mmol (18 mg) of 4-dimethylaminopyridine (DMAP), and place them in a reaction tube with a side arm. Seal the tube, evacuate it under vacuum, and purge with argon. Dissolve 0.71 mmol (72 mg) of 1,3-cyclopentanediol in 10 mL of anhydrous dichloromethane, inject it into the reaction tube, and stir at room temperature under argon for 30 min. Then, add 1.58 mmol (303 mg) of 1-ethyl-(3-dimethylaminopropyl)carbodiimide hydrochloride (EDAC), and continue stirring overnight (about 10 h). After the reaction, add 20 mL of water and stir for 30 min to quench the reaction. Transfer the solution to a separatory funnel, extract with water and dichloromethane twice, and collect the lower dichloromethane layer. Dry with anhydrous magnesium sulfate, and filter to obtain the colorless liquid. Finally, purify the sample by column chromatography and evaporate the solvent under reduced pressure to obtain a white powder sample CPTA. ^1^H NMR (600 MHz, CDCl_3_) δ 5.60 (s, 2H), 2.35 (s, 2H), 2.23 (s, 2H), 2.00 (s, 2H). ^19^F NMR (376 MHz, CDCl_3_) δ -139.04 (m, 4 F), -150.83 (m, 4 F). HRMS (MALDI-TOF) calculated *m/z* [Na]^+^ for C_19_H_8_F_8_N_6_O_4_, 536.0479. Found, 559.0370.

### Process of photolithography patterning

Substrate Pretreatment: The substrates to be patterned (including glass, quartz, silicon wafers, etc.) are sequentially ultrasonically cleaned for 30 min using cleaning solution, deionized water, and isopropyl alcohol (twice). After cleaning, the substrates are soaked in isopropyl alcohol for storage. Before use, they are re-ultrasonically cleaned and dried under a nitrogen flow; Quantum Dot Layer deposition: The quantum dots are dispersed in *n*-octane, with a crosslinker content of 10 w%. Ultrasonic dispersion is used to fully dissolve the QDs. Before use, the solution is filtered, and the QD solution is spin-coated onto the substrate using a spin coater; UV Exposure: The quantum dot-coated substrate is placed under a pre-designed photomask, and exposed to ultraviolet light (254 nm) with a UV dose of 20 mJ cm^−2^; Developing: A non-polar solvent such as *n*-octane is used as the developer. The QD-coated substrate is immersed in the solvent for 1 min to dissolve the quantum dot layer in the areas that were not exposed to UV light.

### Fabrication process of patterned QLED devices

The structure of the patterned QLED device is: Glass/ITO/PEDOT:PSS/PF8Cz/QDs@Crosslinker/PMMA/ZnMgO/Al. First, the ITO-coated glass substrate is sequentially ultrasonically cleaned for 30 min in ITO cleaning solution, deionized water, isopropyl alcohol, and isopropyl alcohol, followed by UV ozone treatment before use. Next, a solution of poly(3,4-ethylenedioxythiophene)/polystyrenesulfonate (PEDOT:PSS, AI 4083) is spin-coated at 5000 rpm for 30 s on the ITO layer, followed by annealing at 150 °C for 30 min. Then, a solution of poly(9,9-di-n-octyl-2,7-fluorene-alt-9-octyl-3,6-carbazole) (PF8Cz, 8 mg mL^−1^ in chlorobenzene) is spin-coated at 4000 rpm for 30 s, and annealed at 160 °C for 30 min. Afterward, a QD/crosslinker solution (QDs at 20 mg mL^−1^, crosslinker at 10 wt%, solvent: *n*-octane) is spin-coated at 3000 rpm for 30 s, and the substrate is left at room temperature for 5 min. The QD layer is then exposed to ultraviolet light (UV dose: 20 mJ cm^−2^) under a pre-designed photomask. Excess quantum dots are removed by spin-coating with *n*-octane, and the substrate is left at room temperature for 10 min. Next, a PMMA solution (2 mg mL^−1^ in acetone) is spin-coated at 5000 rpm for 30 s and annealed at 60 °C for 30 min. Then, a ZnMgO solution is spin-coated at 2000 rpm for 30 s and annealed at 60 °C for 30 min. Finally, an aluminum cathode (100 nm) is deposited in a custom high-vacuum deposition chamber. The entire process, except for the PEDOT:PSS layer, is carried out in a nitrogen-filled glove box.

### Characterization

The compound CPTA was characterized using ^1^H NMR (600 MHz, 293 K) and ^19^F NMR (400 MHz, 293 K), high-resolution mass spectrometry (GCT-MS, Micromass, UK), high-performance liquid chromatography (HPLC), and single-crystal X-ray diffraction analysis. Steady-state absorption spectra were recorded using a Shimadzu UV-3600 UV-VIS-NIR spectrophotometer, while emission spectra were acquired with a Horiba FluoroMax-4-NIR spectrophotometer. Fluorescence lifetimes were measured on an Edinburgh Instruments FLS 1000, equipped with a Xenon lamp, a microsecond flash lamp, and a 375 nm picosecond pulsed laser. The relative fluorescence quantum yields of solutions were determined using the absolute method, employing the integrating sphere attached to the FluoroMax-4-NIR spectrophotometer. To prevent internal filter effects, the absorbance of the solutions was kept below 0.1. FTIR spectra were obtained using Nicolet iS50 FTIR, thermogravimetric analysis was performed with TG 209F3, and transmission electron microscopy (TEM) was conducted with an in-situ aberration-corrected double-spherical TEM (JEM-ARM300F). Patterned images were captured using an Olympus FV3000 equipped with a UV light source.

### Preparation of crystals

The CPTA crystal (CCDC: 2418785) was prepared by slowly evaporating of the 8 mL dichloromethane with hexane (v/v = 4/1) in the 10 mL glass sample bottle at room temperature, respectively.

## Supplementary information

Supplementary Information

## Data Availability

The authors declare that all data supporting the findings of this study can be found within the paper and its Supplementary information files. Additional data supporting the findings of this study are available from the corresponding author (A.W.T. or Z.W.M.) upon reasonable request.
