# Supplementary material for: Rigid crosslinker-assisted nondestructive direct photolithograph for patterned QLED displays
Source: Light Sci Appl. 2025 Jul 24;14:251. doi: 10.1038/s41377-025-01918-7 (PMC12290042; doi:10.1038/s41377-025-01918-7)
Supplement: Supplementary file 1 — Supplementary Information [file 41377_2025_1918_MOESM1_ESM.pdf]

**Supplementary Information for**

# Rigid Crosslinker-Assisted Nondestructive Direct Photolithograph for Patterned QLED Displays

Zhong Chen, Zhongwei Man*, Shichao Rao, Jinxing Zhao, Shuaibing Wang, Runtong Zhang, Feng Teng and Aiwei Tang*

Key Laboratory of Luminescence and Optical Information, Ministry of Education,
School of Physical Science and Engineering
Beijing Jiaotong University
Beijing 100044, P. R. China
E-mail: zwman@bjtu.edu.cn; awtang@bjtu.edu.cn

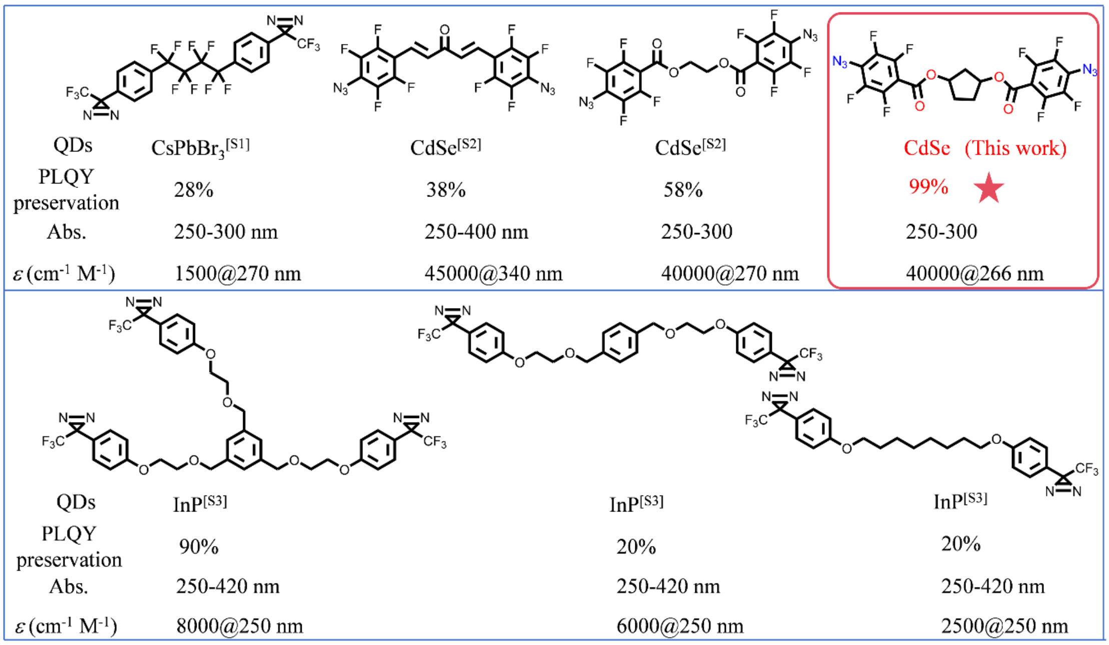


**Fig. S1** PLQY of the selected azide group crosslinker examples for direct photolithography.

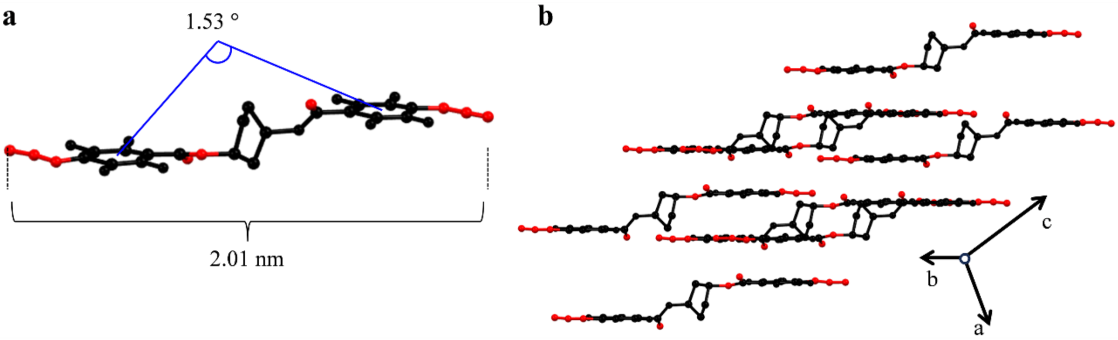


**Fig. S2** a) molecular conformation and b) arrangement of CPTA-crystal

**Tab.S1** Summary of the single crystals data of CPTA crystal.

| | **CPTA-crystal** |
|---|---|
| CCDC | 2418785 |
| Crystal system | triclinic |
| Space group | P-1 |
| a/Å | 6.7412(5) |
| b/Å | 7.4629(5) |
| c/Å | 10.0953(7) |
| α/° | 93.927(5) |
| β/° | 102.665(6) |
| γ/° | 95.306(6) |
| $V$/Å$^3$ | 491.34(6) |
| Z Value | 1 |
| $V$/Z/Å$^3$ | 491.34 |
| $p_{calc}$/g cm$^{-3}$ | 1.813 |
| Final R indexes [I>=2σ (I)] | R1 = 0.0419, wR2 = 0.1124 |
| Final R indexes [all data] | R1 = 0.0448, wR2 = 0.1174 |
| GOF$^C$ | 1.078 |

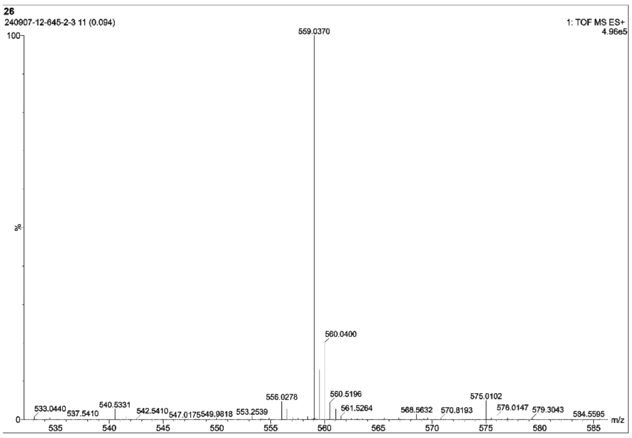


**Fig. S3** HRMS (ESI) m/z [M+Na]$^+$ calcd for $C_{19}H_8F_8N_6O_4$: 536.0479, found: 559.0370 (+ Na$^+$).

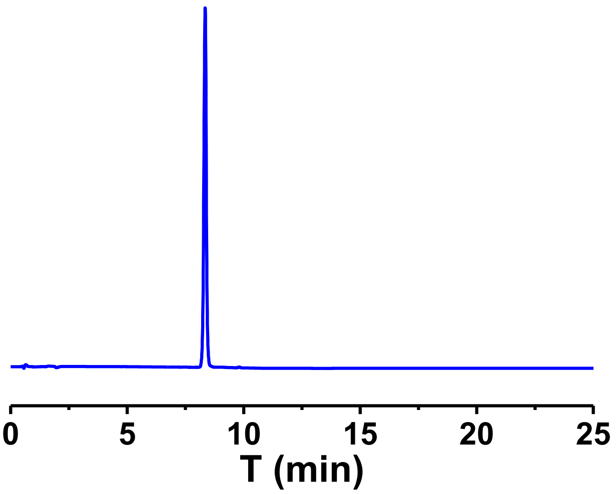


**Fig. S4** HPLC analysis of the CPTA compound.

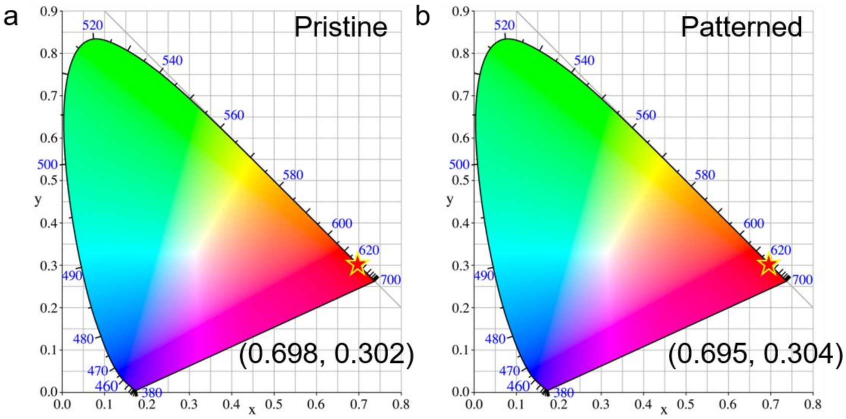


**Fig. S5** CIE coordinates of the **a** pristine and **b** patterned red QDs.

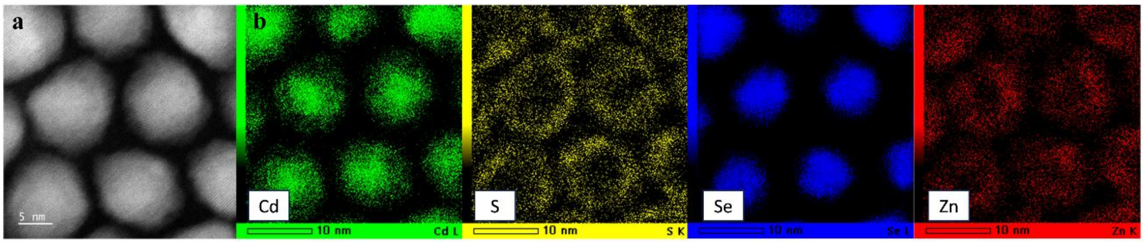


**Fig. S6** **a** HRTEM image and **b** energy dispersive spectroscopy elemental maps of red CdSe QDs before crosslinking.

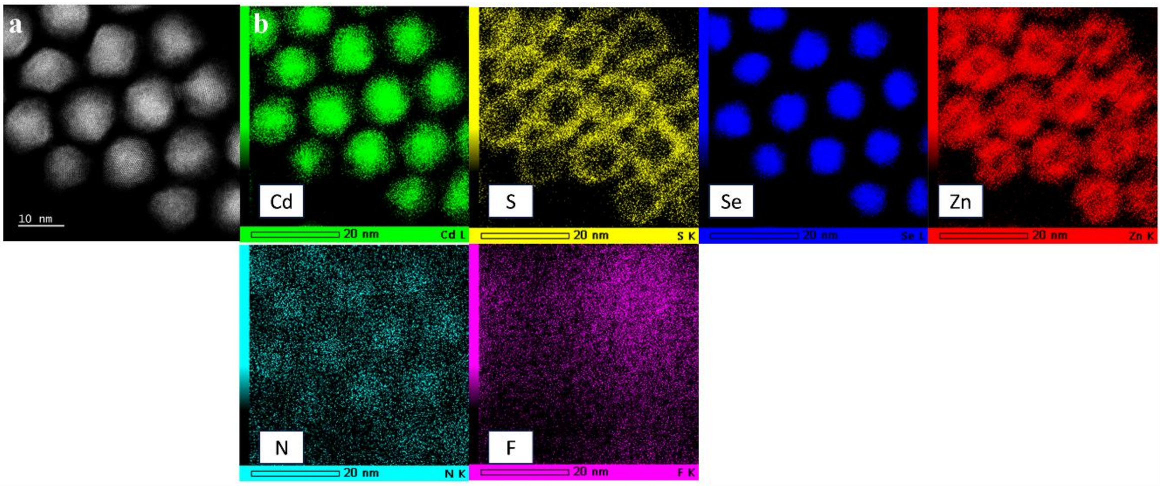


**Fig. S7** **a** HRTEM image and **b** energy dispersive spectroscopy elemental maps of red CdSe QDs after crosslinking.

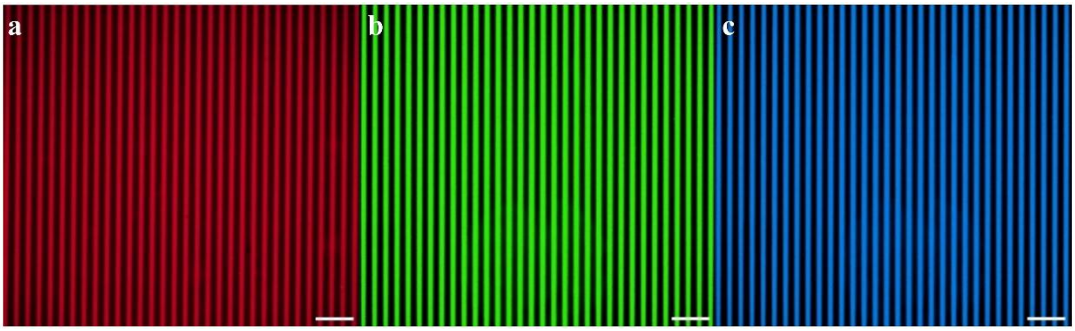


**Fig. S8** Microscopic PL images of **a** red, **b** green, and **c** blue line arrays, respectively. Scale bar: 70 μm.

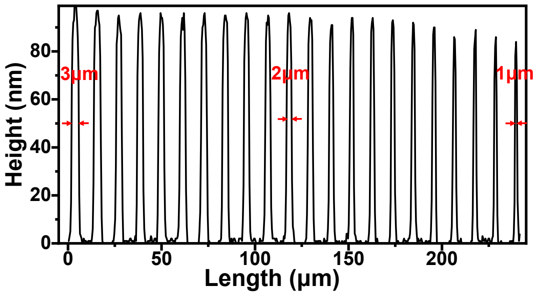


**Fig. S9** Height profile of line patterns.

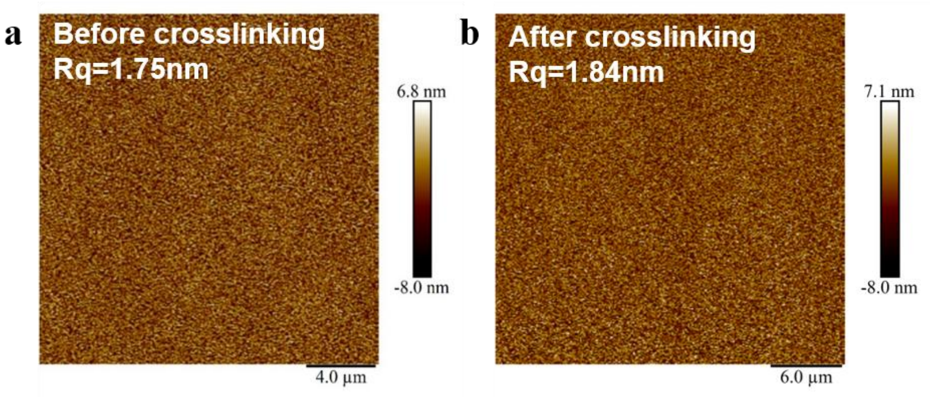


**Fig. S10** The surface roughness analysis of QD films before **a** and after **b** crosslinking.

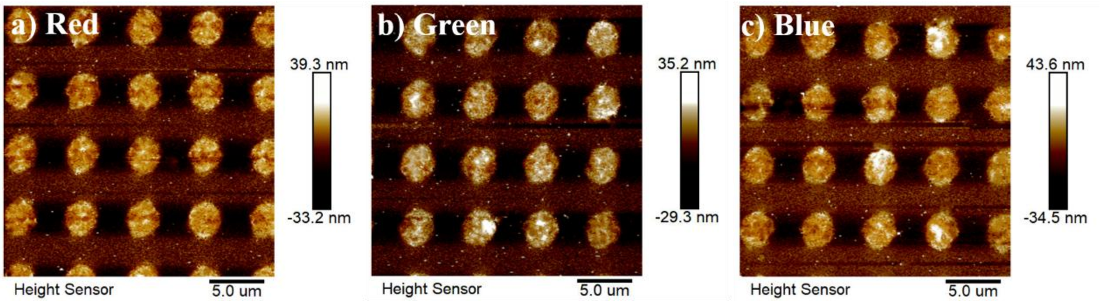


**Fig. S11** AFM images of a) red, b) green and c) blue QD pixel arrays with a width of 2 μm.

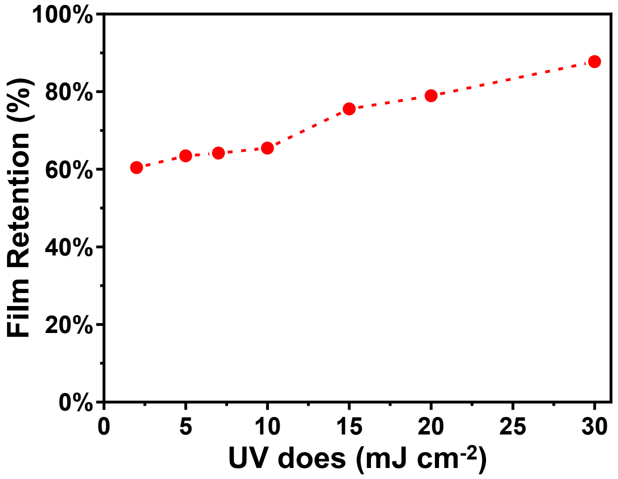


**Fig. S12** The relationship between film retention rate and UV exposure dose.

The calculation formula for the film retention rate is as follows:

$$Film\ Retention=\frac{QDs\ content\ after\ development}{QDs\ content\ before\ development}\times 100\%$$

**Tab. S2** Photo-physical parameters of QD films with CPTA at different doses.

| Sample (mJ cm$^{-2}$) | $\tau_{avg}$ (ns) | $\tau_1$ (ns) | $A_1$ (%) | $\tau_2$ (ns) | $A_2$ (%) | PLQY (%) | $k_r$ (μs$^{-1}$) | $k_{nr}$ (μs$^{-1}$) |
|---|---|---|---|---|---|---|---|---|
| Pristine | 12.02 | 8.61 | 63.85 | 18.17 | 36.15 | 43.96 | 36.57 | 46.62 |
| 20 | 11.83 | 8.13 | 58.87 | 17.12 | 41.13 | 43.52 | 36.78 | 47.74 |
| 50 | 11.26 | 7.23 | 52.90 | 15.79 | 47.10 | 40.64 | 36.09 | 52.71 |
| 70 | 10.99 | 7.00 | 51.89 | 15.30 | 48.11 | 40.22 | 36.59 | 54.39 |
| 100 | 10.65 | 7.40 | 65.46 | 16.82 | 34.54 | 38.42 | 36.07 | 57.82 |
| 150 | 10.34 | 7.02 | 62.49 | 15.85 | 37.51 | 37.46 | 36.22 | 60.48 |

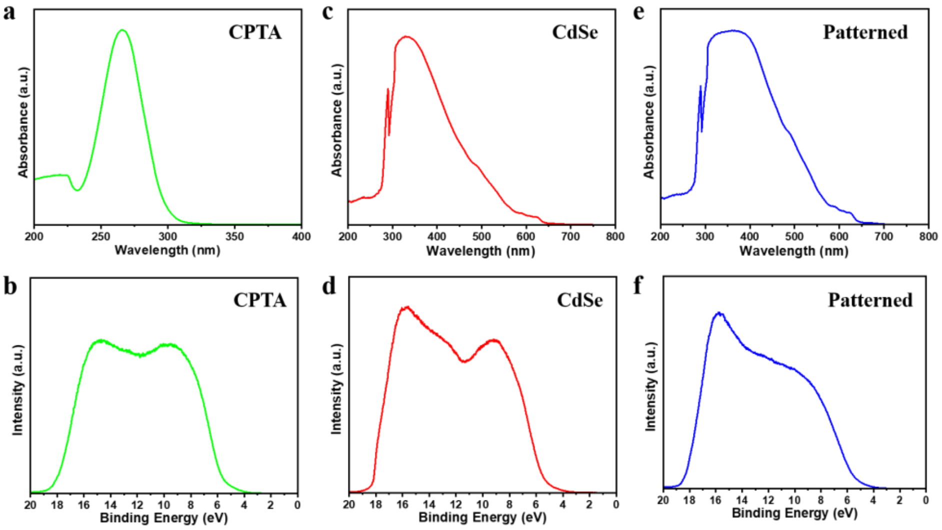


**Fig. S13** **a** Absorption and **b** UPS spectra of CPTA film. **c** Absorption and **d** UPS spectra of CdSe film. **e** Absorption and **f** UPS spectra of patterned film.

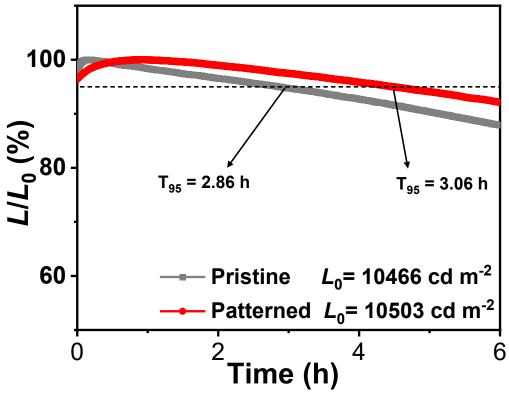


**Fig. S14** Temporal changes in relative luminance $L/L_0$ of pristine and patterned QLED at 10,000 nit.

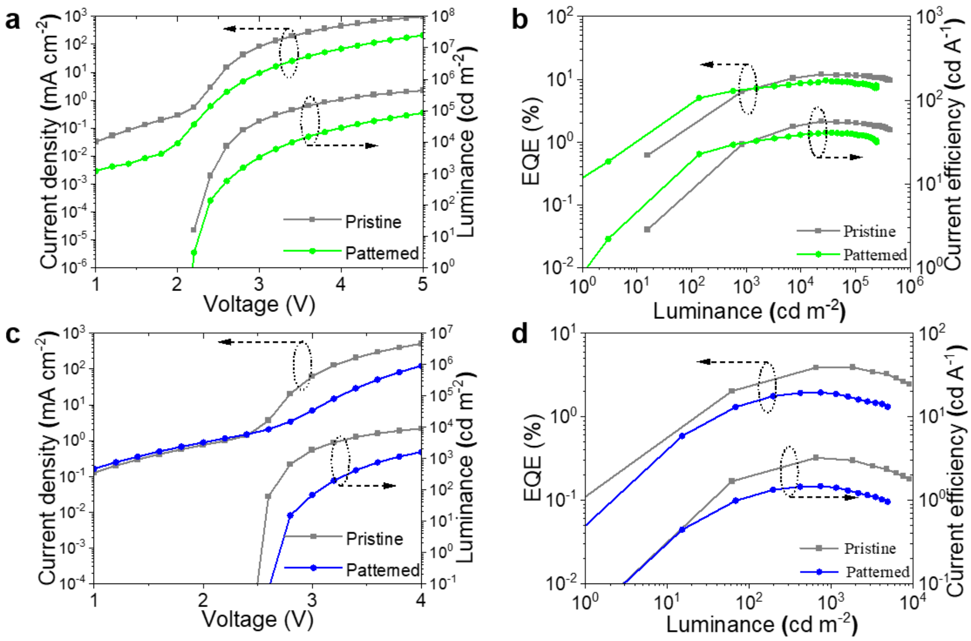


**Fig. S15** **a** *J*-*V*-*L* and **b** EQE-CE-*L* characteristics of pristine and patterned green devices. **c** *J*-*V*-*L* and **d** EQE-CE-*L* characteristics of pristine and patterned blue devices.

## References


1. Liu, D. *et al.* Nondestructive Direct Optical Patterning of Perovskite Nanocrystals with Carbene-Based Ligand Cross-Linkers. *ACS Nano* **18**, 6896–6907 (2024).
2. Lu, S. *et al.* Beyond a Linker: The Role of Photochemistry of Crosslinkers in the Direct Optical Patterning of Colloidal Nanocrystals. *Angewandte Chemie International Edition* **61**, e202202633 (2022).
3. Fu, Z. *et al.* Direct Photopatterning of Colloidal Quantum Dots with Electronically Optimized Diazirine Cross-Linkers. *Journal of the American Chemical Society* **146,** 28895–28905 (2024).
